# Enhanced Insulin Sensitivity Associated with Provision of Mono and Polyunsaturated Fatty Acids in Skeletal Muscle Cells Involves Counter Modulation of PP2A

**DOI:** 10.1371/journal.pone.0092255

**Published:** 2014-03-14

**Authors:** Francesca Nardi, Christopher Lipina, David Magill, Rima Hage Hassan, Eric Hajduch, Alexander Gray, Harinder S. Hundal

**Affiliations:** 1 Division of Cell Signalling and Immunology, Sir James Black Centre, College of Life Sciences, University of Dundee, Dundee, United Kingdom; 2 Institut National de la Santé et de la Recherche Médicale (INSERM), Centre de Recherche des Cordeliers, UMR-S 872, Paris, France; 3 Université Pierre et Marie Curie – Paris 6, UMR-S 872, Paris, France; 4 Université Paris Descartes, UMR-S 872, Paris, France; GDC, Germany

## Abstract

**Aims/Hypothesis:**

Reduced skeletal muscle insulin sensitivity is a feature associated with sustained exposure to excess saturated fatty acids (SFA), whereas mono and polyunsaturated fatty acids (MUFA and PUFA) not only improve insulin sensitivity but blunt SFA-induced insulin resistance. The mechanisms by which MUFAs and PUFAs institute these favourable changes remain unclear, but may involve stimulating insulin signalling by counter-modulation/repression of protein phosphatase 2A (PP2A). This study investigated the effects of oleic acid (OA; a MUFA), linoleic acid (LOA; a PUFA) and palmitate (PA; a SFA) in cultured myotubes and determined whether changes in insulin signalling can be attributed to PP2A regulation.

**Principal Findings:**

We treated cultured skeletal myotubes with unsaturated and saturated fatty acids and evaluated insulin signalling, phosphorylation and methylation status of the catalytic subunit of PP2A. Unlike PA, sustained incubation of rat or human myotubes with OA or LOA significantly enhanced Akt- and ERK1/2-directed insulin signalling. This was not due to heightened upstream IRS1 or PI3K signalling nor to changes in expression of proteins involved in proximal insulin signalling, but was associated with reduced dephosphorylation/inactivation of Akt and ERK1/2. Consistent with this, PA reduced PP2Ac demethylation and tyrosine^307^phosphorylation - events associated with PP2A activation. In contrast, OA and LOA strongly opposed these PA-induced changes in PP2Ac thus exerting a repressive effect on PP2A.

**Conclusions/Interpretation:**

Beneficial gains in insulin sensitivity and the ability of unsaturated fatty acids to oppose palmitate-induced insulin resistance in muscle cells may partly be accounted for by counter-modulation of PP2A.

## Introduction

Sustained elevation in circulating non-esterified free fatty acids (NEFA) as seen during obesity and Type 2 diabetes is associated with impaired insulin signalling in tissues such as skeletal muscle thus potentially contributing to disturbances in whole body glucose metabolism in obese or diabetic individuals [1]–[3]. In particular, over-provision of saturated fatty acids (SFA, e.g. palmitate) result in tissue accumulation of lipotoxic fatty acid derivatives such as diacylglycerol (DAG) and ceramide that promote activation of (i) DAG-sensitive PKCs, (ii) atypical PKCs and (iii) PP2A that, in turn, impair IRS- and Akt-directed insulin signalling by mechanisms involving IRS serine phosphorylation or repression of Akt activation/phosphorylation [4]–[9]. Strikingly, dietary substitution of SFAs or co-supplementation with unsaturated fatty acids antagonise the insulin desensitising effects of SFAs, enhance energy expenditure and improve muscle lipid composition and serum acylcarnitine profiles in humans [10]–[14]. Such observations are in line with cell-based studies demonstrating that monounsaturated fatty acids (MUFAs), such as palmitoleate and oleate, not only attenuate the SFA-induced loss in mitochondrial integrity and function but also repress their proinflammatory and ER stress-inducing potential [15]–[18]. Moreover, the observation that increases in serum palmitoleate induce a strong insulin potentiating effect in mouse liver and skeletal muscle implies that this MUFA possesses unique metabolic attributes not held by its saturated counter-part, palmitate [19]. However, despite the observed gain in insulin sensitivity brought about by palmitoleate in these peripheral tissues, our understanding of the underlying mechanism remains poor [19].

In this study we have investigated the effects of oleic acid (OA; a *cis*-C18:1, n-9 MUFA), and linoleic acid (LOA; a *cis*-C18:2, n-6 PUFA) upon insulin signalling events in cultured rat and human skeletal myotubes. Our work reveals that both oleic acid and linoleic acid induce an increase in insulin sensitivity based on enhanced Akt- and Erk-directed insulin signalling. These effects cannot be attributed to an elevation in upstream IRS/PI3K signalling but were associated with increased tyrosine phosphorylation (Tyr^307^) and demethylation of the PP2A catalytic subunit (PP2Ac) – covalent changes that help retain the phosphatase in a repressed state [20], [21]. In contrast, palmitate (PA) attenuated tyrosine phosphorylation and demethylation of PP2Ac by insulin in line with the known stimulatory effect that this SFA has upon PP2A [22]. These PA-induced changes in PP2A were antagonised by both OA and LOA. Our data indicate that increases in insulin sensitivity brought about by exposure of muscle cells to OA and LOA may, in part, be brought about by covalent modification and cellular repression of PP2A.

## Materials and Methods

### Materials

α-minimal essential medium, fetal bovine serum and antibiotic/antimycotic solution were from Life Technologies. All other reagent-grade chemicals, including BSA and fatty acids, were obtained from Sigma–Aldrich unless otherwise stated. Wortmannin and okadaic acid were from Calbiochem-Merck. Fraction V fatty acid-free BSA was from Roche. Insulin was from R&D systems. Complete protein phosphatase inhibitor tablets were purchased from Roche Diagnostics. Phospho-Akt^Thr308^, phospho-Akt^Ser473^, phospho-ERK1/2^Thr202/Tyr204^, phospho-GSK3^Ser21/9^, phospho-CREB^Ser133^, phospho-Src^Tyr416^, anti-Akt and anti-ERK1/2 antibodies were from Cell Signaling Technology. Anti-GSK3 and anti-PTEN antibodies were from Santa Cruz Biotechnology. Anti-IR (insulin receptor), anti-p85, anti-IRS1 and anti-PP2A subunit C, demethylated were from Millipore. Anti-β actin and anti-GAPDH were from Sigma. Phospho-PP2A^Y307^ was from Abcam.

### Cell culture, fatty acid treatment and cell lysis

Human skeletal muscle cells derived from biopsies (quadriceps muscle) from healthy adult lean volunteers obtained in the context of approved preclinical and clinical trials [23], and *via* the Tissue Bank for Research (Myobank) of the French Association against Myopathies (AFM), in agreement with the French bioethical law on informed consent. Rat L6 muscle cells were originally sourced from Dr Amira Klip (Hospital For Sick Children, Toronto) [24].

Rat L6 and human skeletal myotubes were cultured as described previously [25], [26]. Myotubes were exposed to serum free media containing 2% (w/v) fatty acid-free BSA alone (control vehicle) or fatty acids that had been preconjugated to BSA (2% w/v) at concentrations and for the times indicated in figure legends. In some experiments myotubes were incubated with insulin (10 min) at concentrations indicated in figure legends. At the end of appropriate experimental treatments cells were washed using cold phosphate-buffered saline and lysed using lysis buffer [50 mM Tris/HCl, pH 7.4, 0.27 M sucrose, 1 mM sodium orthovanadate, 1 mM EDTA, 1 mM EGTA, 10 mM sodium 2-glycerophosphate, 50 mM sodium fluoride, 5 mM sodium pyrophosphate, 1% (w/v) Triton X-100, 0.1% (v/v) 2-mercaptoethanol and protease inhibitor (one tablet/50 ml)]. Whole-cell lysates were centrifuged for 10 min at 6000 rpm, 4°C and supernatant frozen in liquid nitrogen prior to storage at -20°C. Protein concentration was measured using the Bradford assay [27].

### Immunoblotting, IRS-1 immunoprecipitation and analysis of cellular PIP_3_

Proteins from cell lysates (30 μg) were separated by SDS-PAGE and the abundance/activation status of specific proteins assessed by immunoblotting [25] using primary antibodies to proteins of interest followed by exposure to an appropriate peroxidase-conjugated IgG for 1h at room temperature. Immunoreactive bands were detected by enhanced chemiluminescence on Kodak X-OMAT film and quantified using Image J software (http://rsbweb.nih.gov/ij/). Analysis of p85-PI3K association with IRS1 and cellular PIP_3_ content were respectively assessed by coimmunoprecipitation with IRS1 and use of a sensitive time-resolved fluorescence resonance energy transfer (FRET)-based assay as described previously [28], [29].

### Statistical analyses

One-way ANOVA or a Student's *t* test as appropriate was performed using GraphPad Prism software with differences considered statistically significant at *P*<0.05.

## Results

### Effects of OA and LOA on insulin signalling

**Figure 1:**
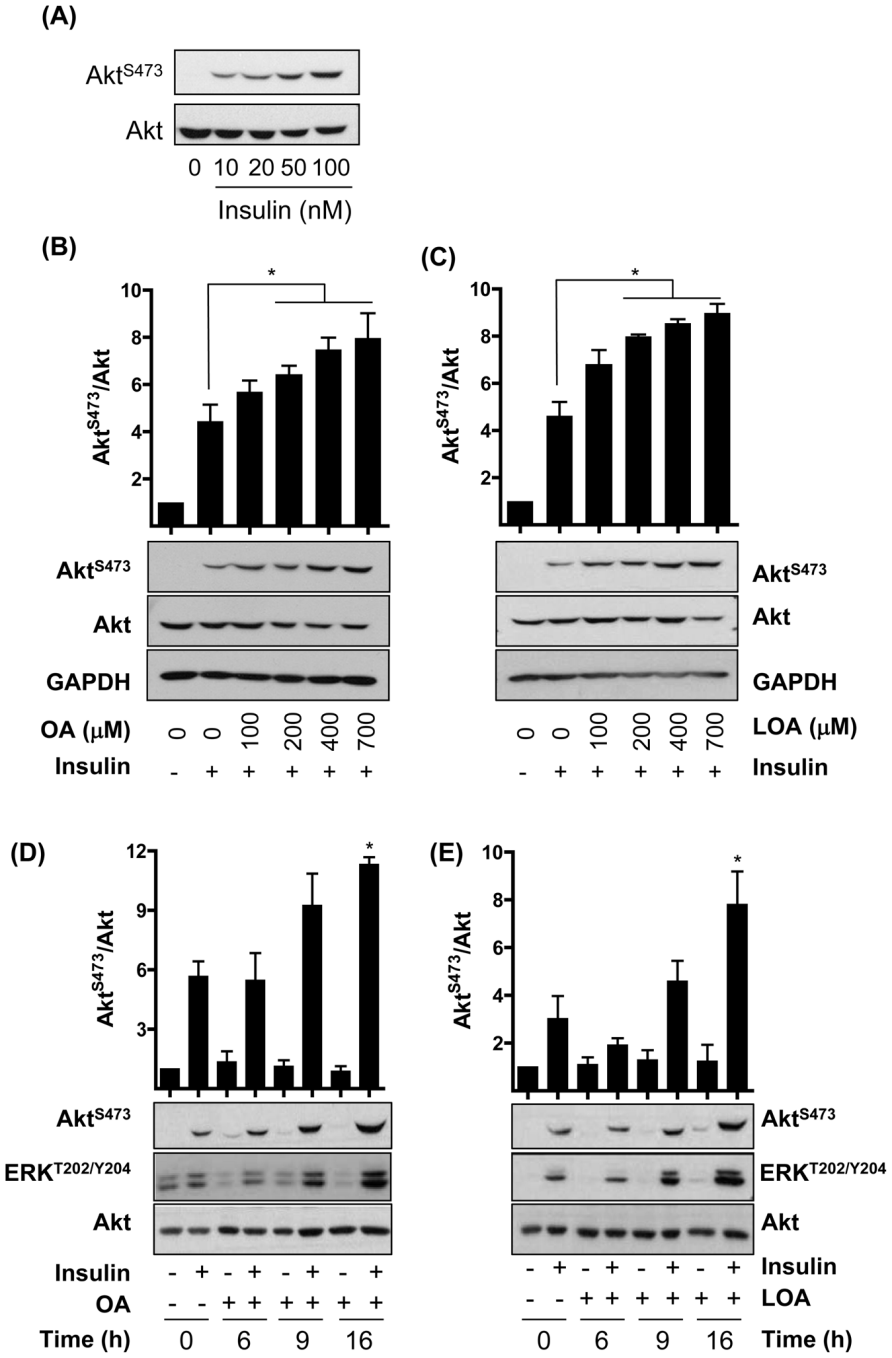
Effects of insulin, oleic acid (OA) and linoleic acid (LOA) on Akt and ERK phosphorylation in rat L6 myotubes. (**A**) L6 myotubes were serum-starved for 2h prior to stimulation with insulin (10 min) at concentrations indicated. (**B,C**) L6 myotubes were incubated with serum-free media containing 2% (w/v) BSA (vehicle) ± (**B**) oleic acid (OA) or (C) linoleic acid (LOA) at concentrations indicated, for 16h prior to stimulation with insulin (20 nM, 10min). (D,E) L6 myotubes were incubated as in (**B**) ± 700 μM (**D**) OA or (**E**) LOA for times indicated, prior to stimulation with insulin (20 nM, 10min). (A-E) Cell lysates were immunoblotted using antibodies against the proteins indicated. The asterisks signify a significant change between the indicated bars (p<0.05).

To characterise any insulin potentiating effect that OA and LOA may have on intracellular signalling we performed time and dose studies for both NEFAs but in the presence of a sub-maximal (20 nM) insulin concentration (Fig 1A). At this concentration, insulin induced a modest increase in Akt phosphorylation, which was barely detectable in untreated cells (Fig 1B,1C). However, Akt phosphorylation was enhanced significantly when myotubes were preincubated with OA or LOA for 16h in a dose-dependent manner. This enhancement was significant at fatty acid concentrations as low as 200 μM (i.e. within the physiological range) and was ∼2-fold greater than that elicited by insulin treatment alone when myotubes were pre-exposed to maximally effective doses (700 μM) of OA or LOA (Fig 1B, 1C). The increase in Akt phosphorylation was detectable after 9h but was not maximal until 16h of fatty acid treatment (Fig 1D,1E). Extended fatty acid incubation periods up to 24h did not induce further increases in Akt phosphorylation over and above that seen after 16h (data not shown). Fig 1D and 1E show that the increase in signalling was not restricted to Akt and that ERK1/2 phosphorylation was also enhanced. In the absence of insulin, neither fatty acid had any effect on Akt or ERK1/2 phosphorylation (Fig 1D, 1E). It is also important to stress that treatment of myotubes with the vehicle (i.e. BSA) alone did not enhance insulin-dependent phosphorylation of Akt or ERK when compared with cells not exposed to the vehicle (data not shown).

**Figure 2:**
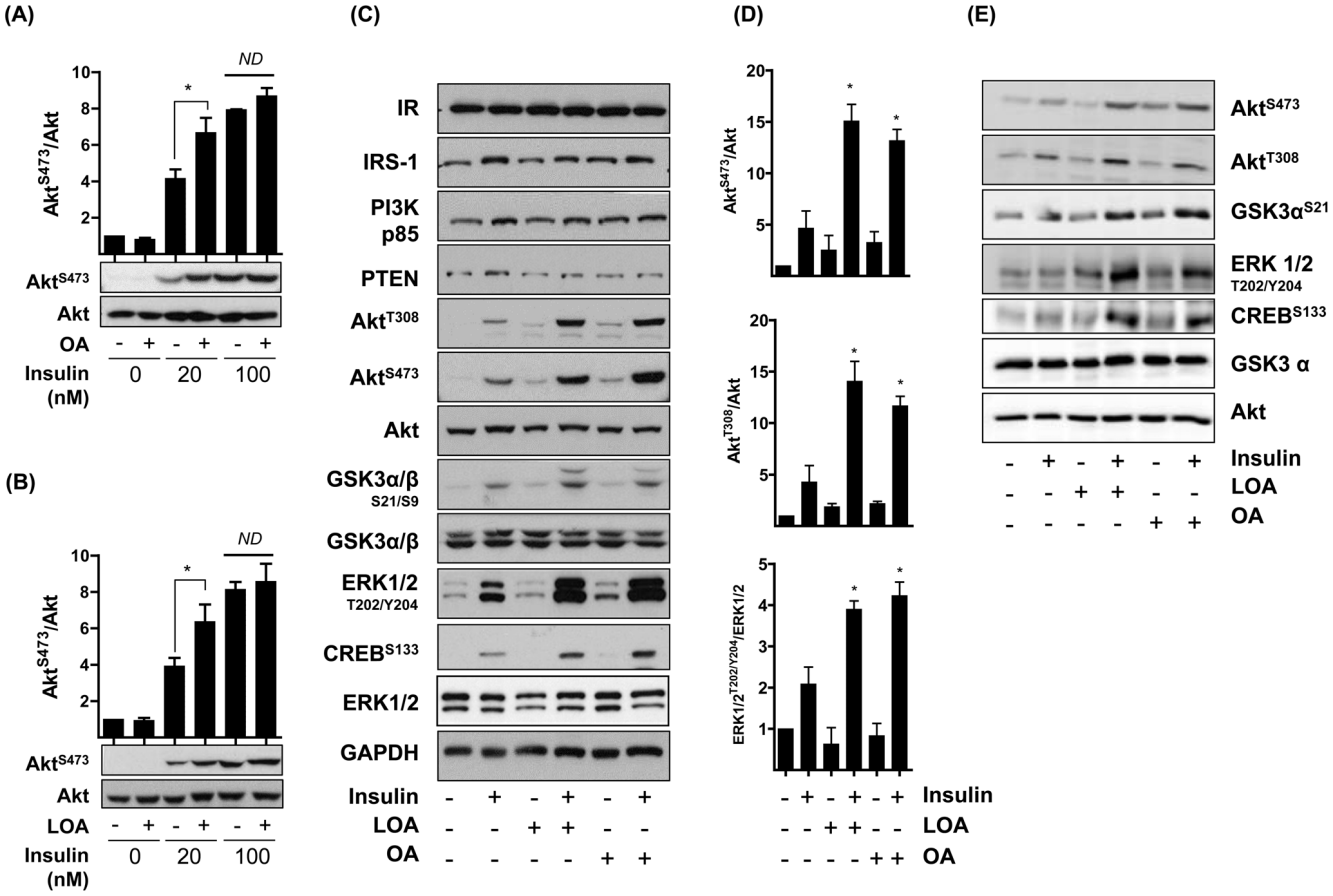
Oleic acid (OA) and linoleic acid (LOA) enhance insulin sensitivity of rat L6 and primary human myotubes. (**A**) L6 myotubes were incubated with serum-free media containing 2% (w/v) BSA (vehicle) ± 700 μM (A) oleic acid (OA) or (B) linoleic acid (LOA) for 16h prior to stimulation with insulin at concentrations indicated (10min). (**C, D**) L6 myotubes were incubated as in (A) prior to stimulation with insulin (20 nM, 10min). (**E**) Human myotubes were incubated with 200 μM OA or LOA for 24h prior to stimulation with insulin (20 nM, 10min). Cell lysates from A-E were subsequently immunoblotted using antibodies against the proteins indicated with quantification of phospho-Akt (Ser473 and Thr308) and phospho-ERK1/2 shown in panel A,B and D expressed as mean ± SEM of at least three experiments. The immunoblots shown in panel E are representative of two separate experiments. The asterisks signify a significant change (p<0.05) between the indicated bars (A,B) or the insulin-treatment alone (D).

The increased sensitisation towards insulin elicited by both NEFAs was most apparent and significant when monitored using a sub-maximal insulin dose as no additional increase in Akt phosphorylation was observed when OA- and LOA-treated myotubes were treated with a maximally effective (100 nM) insulin concentration (Fig 2A, 2B). Fig 2C shows that we observe no major changes in the expression of the insulin receptor β-subunit, IRS-1, PI3K-p85 subunit or of 3′-phosphonositide lipid phosphatase (PTEN), all of which lie upstream of Akt and ERK1/2 in response OA or LOA treatment. Furthermore, neither OA nor LOA had any effect on total Akt or ERK1/2 abundance thus excluding this as a possible explanation for the net increase in their phosphorylation. The data highlights that in addition to promoting Ser^473^ phosphorylation, both OA and LOA induce an equivalent gain in Akt-Thr^308^ phosphorylation (Fig 2C, 2D). Equimolar phosphorylation of both sites is required for full activation of Akt and consistent with this we observed elevated phosphorylation of GSK3; a physiological downstream Akt target (Fig 2C). Likewise, enhanced ERK1/2 activation promotes greater phosphorylation/activation of CREB, a transcription factor regulated downstream of the ERK pathway. The increase in insulin-stimulated Akt phosphorylation induced by OA and LOA was not restricted to L6 myotubes. Fig 2E shows that incubation of human skeletal myotubes with OA or LOA caused a similar enhancement in Akt and ERK1/2 phosphorylation in response to insulin and that of GSK3 and CREB, which respectively lie downstream of Akt and ERK signalling.

**Figure 3:**
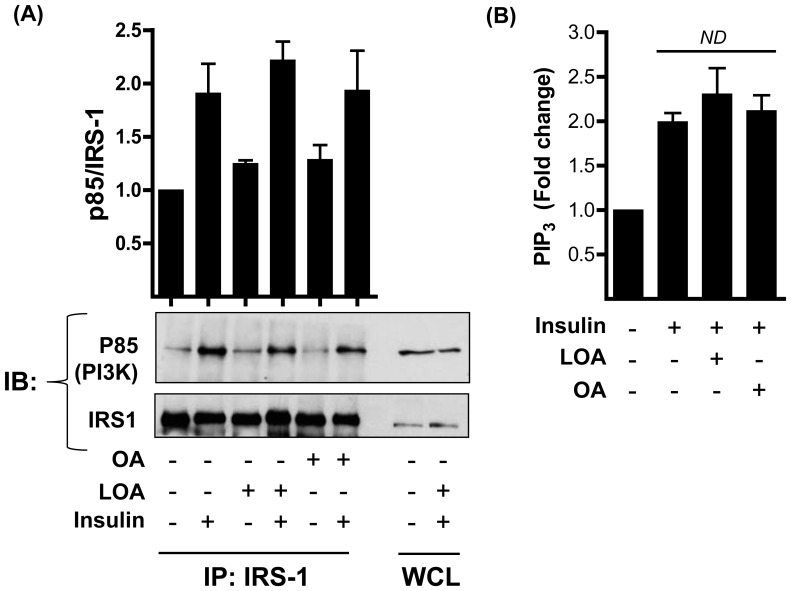
Effects of insulin, oleic acid (OA) and linoleic acid (LOA) on p85/IRS1 association and cellular PIP3 generation in L6 myotubes. (**A,B**) L6 myotubes were incubated with serum-free media containing 2% (w/v) BSA (vehicle) ± 700 μM (A) oleic acid (OA) or (**B**) linoleic acid (LOA) for 16h prior to stimulation with insulin at concentrations indicated (10min). (**A**) IRS-1 was immunoprecipitated from whole cell lysates (WCL) using an anti-IRS-1 antibody. The immunopellet was probed with an anti-p85 antibody. (**B**) Cells were treated as in (A) lysed and assayed for PIP_3_ content [28]. The bar graph values (A & B) are presented as mean ± SEM of three separate experiments.

To understand how OA and LOA might enhance insulin-signalling we monitored the effect of both fatty acids on p85-PI3-kinase association with IRS1 and cellular PIP_3_ synthesis. As anticipated, based on coprecipitation analysis, Fig 3A shows that insulin induced p85-PI3K interaction with IRS1. This interaction was not influenced by OA or LOA. Since Akt activation relies upon insulin-dependent PIP_3_ synthesis it is possible that OA and LOA may have enhanced production of this lipid. However, Fig 3B shows that, irrespective of whether muscle cells were incubated in the absence or presence of OA or LOA, insulin stimulated PIP_3_ synthesis by ∼2-fold.

### OA and LOA help sustain Akt and ERK1/2 activation

**Figure 4:**
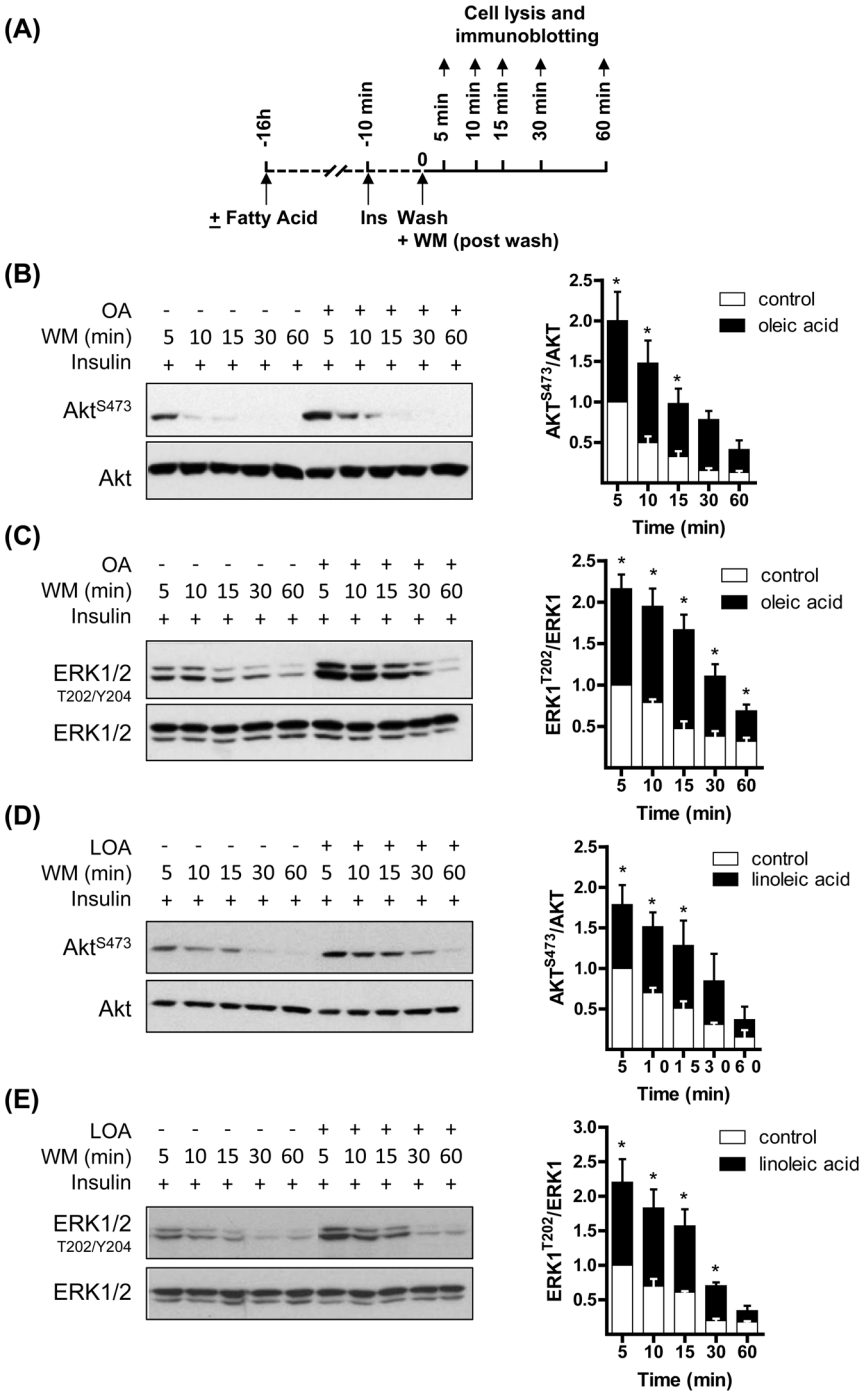
Oleic acid (OA) and linoleic acid (LOA) enhance and prolong insulin-dependent phosphorylation of Akt and ERK in L6 myotubes. The experimental approach used to test the effect of OA and LOA provision on Akt and ERK1/2 phosphorylation is depicted in (A). L6 myotubes were incubated with serum-free media containing 2% (w/v) BSA (vehicle) ± 700 μM (B and C) oleic acid (OA) or (D and E) linoleic acid (LOA) for 16h prior to stimulation with insulin (20 nM, 10min). Cells were washed to remove insulin and subsequently incubated with 100 nM wortmannin (WM) and lysed at times indicated. Immunoblots and relevant quantification of phospho-Akt and phospho-ERK1/2 in L6 myotubes incubated with OA (B,C) or LOA (D,E). Data in the bar graphs is presented as mean ± SEM (n = 3) with asterisks indicating a significant change (p<0.05) between the respective filled (fatty acid treatment) and unfilled (control) bar at each time point.

Given that signalling events at the level of IRS1/PI3K were unaffected by OA or LOA we investigated the possibility that increased phosphorylation/activation of Akt and ERK1/2 by insulin may be a consequence of their reduced dephosphorylation. L6 myotubes were incubated in the absence or presence of these fatty acids for 16h and subjected to an acute insulin challenge in the penultimate 10 min period of this fatty acid incubation. Myotubes were subsequently washed to remove extracellular insulin and reincubated in media with 100 nM wortmannin (to inhibit PI3K and any PIP_3_ synthesis still occurring following removal of insulin) for up to 60 min post-wash. Muscle cells were lysed at time points indicated in Fig 4A and Akt and ERK1/2 phosphorylation assessed in cell lysates. In the absence of OA or LOA treatment, Akt and ERK1 were rapidly dephosphorylated upon removal of insulin and returned to baseline by between 15-30 min. However, both kinases retained a greater level of phosphorylation (∼2-fold at each post-wash time point) in myotubes pretreated with OA or LOA (Fig 4B-E). Analysis of the t_1/2_ with which Akt was dephosphorylated revealed that this was increased from 13 min (untreated cells) to 18 min and 25 min in cells that had been preincubated with OA or LOA, respectively. Similarly, the t_1/2_ with which ERK1/2 become desphosphorylated following cell incubation with OA and LOA was increased to 20 min and 25 min respectively, when compared with untreated cells (12 min).

**Figure 5:**
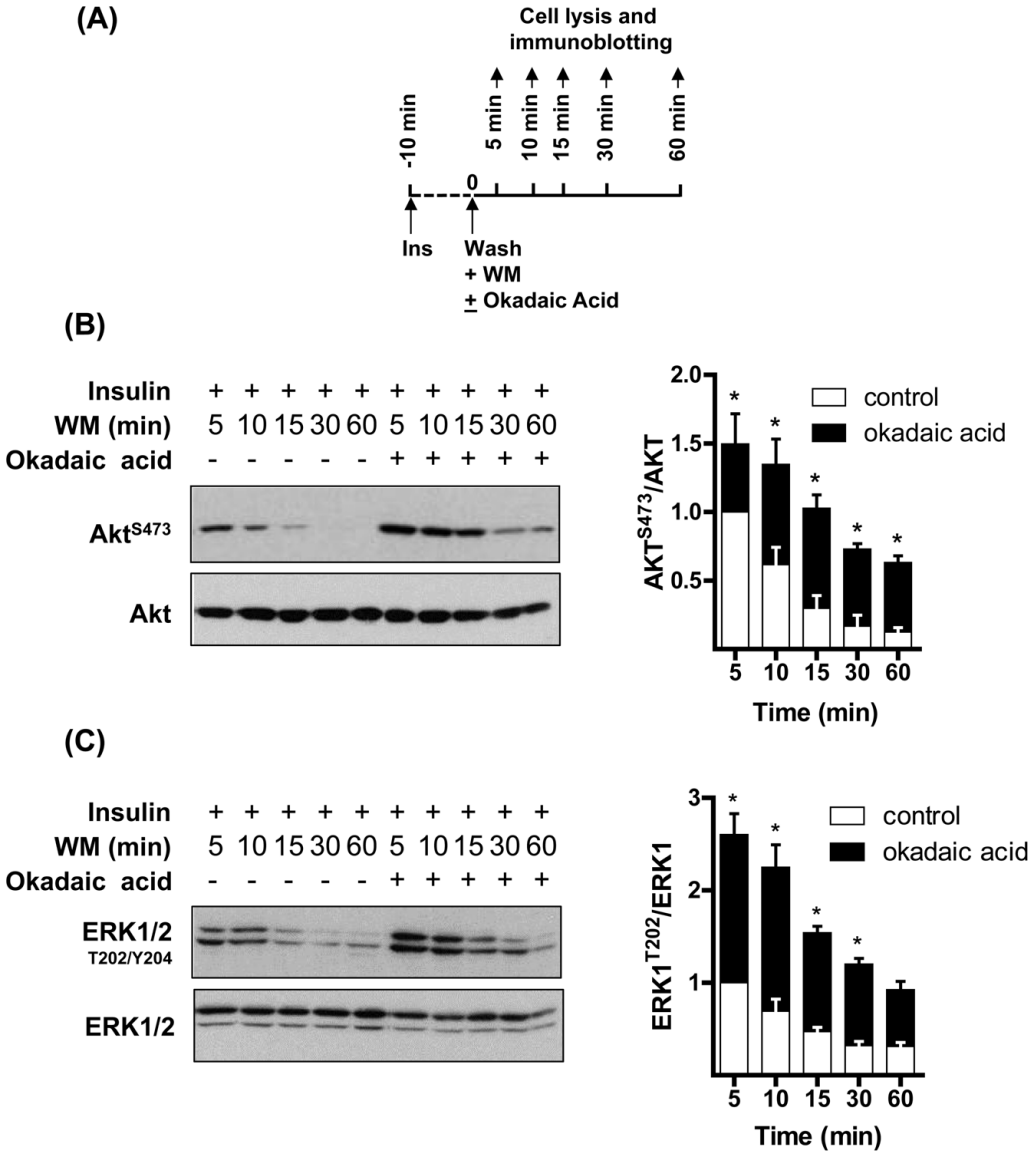
Okadaic acid emulates the insulin-sensitising effect of oleic acid (OA) and linoleic acid (LOA) upon Akt and ERK phosphorylation in L6 myotubes. L6 myotubes were incubated with serum-free media containing okadaic acid (100 nM) or vehicle (dH_2_O) for 15 min and with insulin (20 nM) during the penultimate 10 min period of this incubation. Cells were washed and then maintained in serum-free media containing wortmannin (WM, 100 nM) and lysed at times indicated. Cell lysates were subsequently immunoblotted using antibodies against the proteins indicated. Data in the bar graphs is presented as mean ± SEM (n = 3) with asterisks indicating a significant change (p<0.05) between the respective filled (okadaic acid treatment) and unfilled (control) bar at each time point.

Both Akt and ERK1/2 are physiological targets for protein phosphatase 2A (PP2A, [30], [31]). We hypothesized that inhibiting PP2A using okadaic acid should emulate the effect that we observe following exposure to OA or LOA. Using a similar strategy to that shown in Fig 4A, L6 myotubes were pretreated with okadaic acid (or vehicle (dH_2_O) alone) for 15 min and with insulin in the penultimate 10 min period prior to washing myotubes free from okadaic acid or insulin and reincubation in media containing wortmannin for up to 60 min post-wash. Cells were lysed at time points specified in Fig 5A for analysis of Akt^S473^ and ERK1/2^T202/Y204^ phosphorylation and reveals that pharmacological inhibition of PP2A induces heightened and sustained phosphorylation of both kinases (Fig 5B and 5C) in a manner comparable to that seen following fatty acid treatment (Fig 4B-E).

### PP2Ac phosphorylation and demethylation

**Figure 6:**
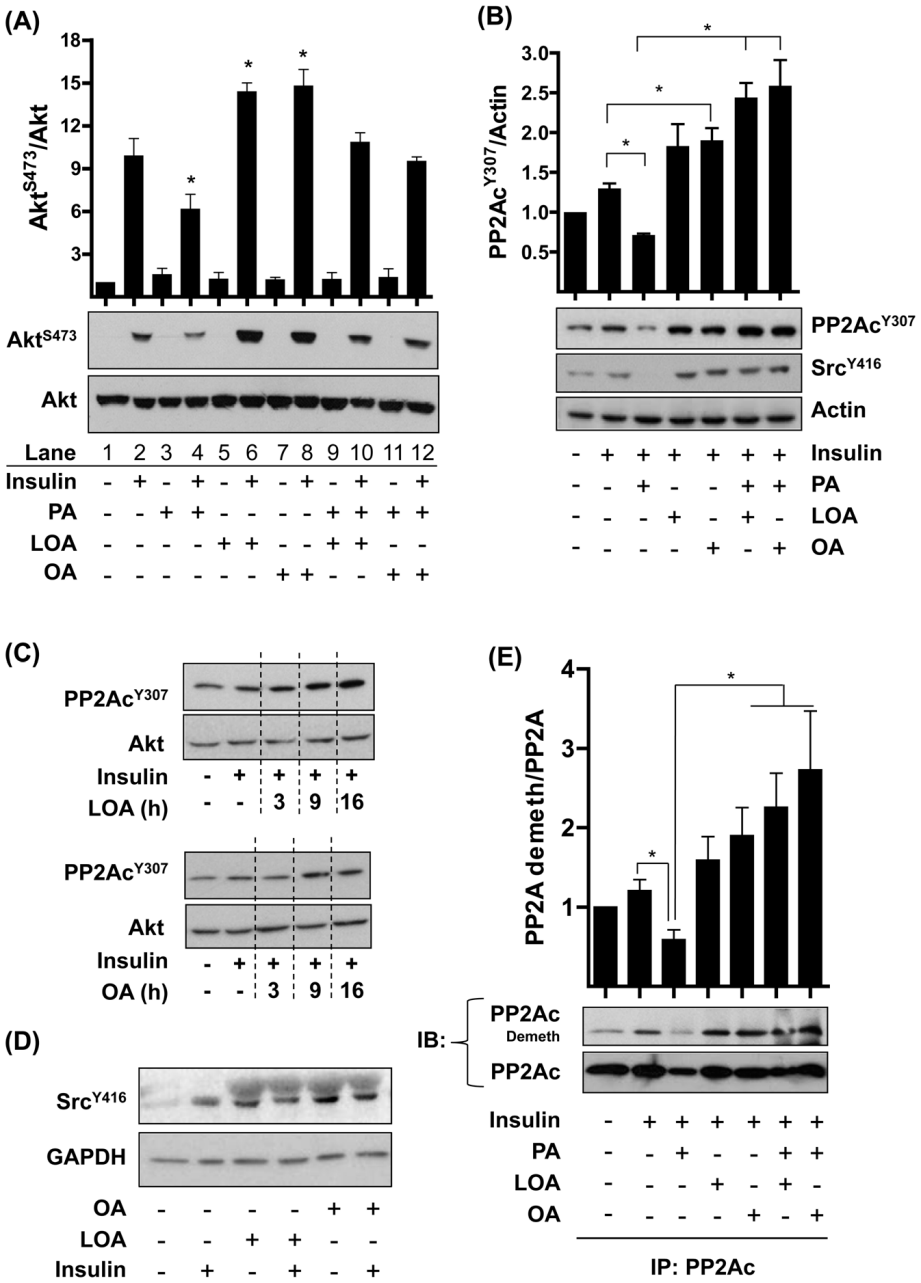
Effects of insulin, palmitate (PA), oleic acid (OA) and linoleic acid (LOA) on Akt, Src and PP2Ac phosphorylation and PP2Ac demethylation in L6 myotubes. L6 myotubes were incubated with serum-free media containing 2% (w/v) BSA (vehicle) ± 700 μM palmitate (PA), oleic acid (OA) or linoleic (LOA) for 16h or for times indicated. In some experiments, cells were treated with fatty acids either alone or in combination as indicated or stimulated with insulin (20 nM, 10min) as indicated (**A-E**). Cell lysates were immunoblotted using antibodies against the proteins/phospho-proteins indicated. In (**E**) PP2Ac was immunoprecipitated from cell lysates and the immunopellet probed with an antibody recognising demethylated PP2Ac. Bar graphs values (A, n = 3; B, n = 4 and E, n = 7) are Mean ± SEM. The asterisks in (**A**) signify a significant change (p<0.05) compared to the insulin treated value (Lane 2) or between the indicated bars (**B & E**).

The above findings support the idea that OA and LOA may enhance insulin signalling *via* repression of PP2A. To assess this possibility we investigated the effect of OA, LOA and the SFA, palmitate, on PP2A. Akt-directed insulin signalling is suppressed by PA and this effect is partly reliant upon the known stimulatory effect that PA exerts upon PP2A [32]. Fig 6A shows that, unlike OA or LOA, sustained exposure of L6 myotubes to PA reduced insulin-stimulated Akt^S473^ phosphorylation (compare lane 2 with 4 and also lanes 6 and 8 with lane 4). This reduction appears to be antagonised when PA-treated myotubes were co-incubated with OA or LOA (compare Lane 4 with lanes 10 and 12). However, the overall gain in phosphorylation remains significantly lower when compared to myotubes exposed to OA or LOA alone (compare lanes 6 and 8 with 10 and 12, respectively).

To assess whether PP2A may be regulated in a manner that accounts for changes in Akt phosphorylation seen in response to insulin, PA, OA and LOA (Fig 6A) we assessed tyrosine^307^ phosphorylation and carboxy-methylation of PP2Ac – two different covalent modifications known to impact on PP2A holenzyme assembly and PP2A activity [21]. Fig 6B shows that compared to untreated cells, insulin was able to acutely induce PP2AcY^307^ phosphorylation by 35%, but that this was negated in myotubes preincubated with PA. In contrast, cellular pre-treatment with OA or LOA not only induced a significant enhancement in PP2AcY^307^ phosphorylation in response to insulin, but blocked the reduction caused by PA. The ability of OA and LOA to enhance PP2AcY^307^ phosphorylation in response to insulin is not an acute event, but requires at least 9h of cell treatment with either fatty acid to manifest (Fig 6C) and thus is consistent with the time course over which there is a detectable increase in insulin sensitivity as judged on the basis of Akt and ERK phosphorylation (Fig 1D and 1E). It is also noteworthy, that this fatty acid-induced increase in PP2AcY^307^ phosphorylation is not restricted to cultured rat muscle cells as we also observe this modulation in primary human muscle cells (data not shown). Src has been implicated as the tyrosine kinase that phosphorylates PPA2cY^307^ and consistent with this insulin, LOA and OA all promoted Src activation based on phosphorylation of its Y^416^ site (Fig 6B). It is noteworthy that Src^Y416^ phosphorylation can be induced independently by insulin and both OA and LOA, although the latter appear slightly more potent. There appears to be no additional gain in phosphorylation of this site if myotubes are co-treated with insulin and OA or LOA (Fig 6D). Analysis of immunoprecipitated PP2Ac with an antibody detecting the demethylated (inactive) protein revealed that insulin enhances PP2Ac demethylation, but that this was abrogated in PA-treated myotubes (Fig 6E). In contrast, both OA and LOA enhanced accumulation/detection of demethylated PP2Ac that occurs with insulin and, when coincubated with PA, repress the anti-demethylation effect of the SFA.

## Discussion

Numerous studies have demonstrated that increased availability of SFAs negatively regulate skeletal muscle insulin sensitivity with important knock-on consequences for processes such as gene expression and cell metabolism that are normally regulated by the hormone [8], [33]–[35]. In contrast, considerable evidence supports the view that insulin sensitivity is positively correlated with supply of MUFAs and PUFAs and that, in many instances, their provision not only promotes beneficial metabolic changes but negate some of the adverse effects associated with SFA oversupply [18], [35], [36]. Indeed, previous work from our group has demonstrated that, unlike PA, provision of MUFAs (e.g. palmitoleate and oleate) can induce insulin-like effects upon distal cellular responses, such as glucose uptake by inducing plasma membrane recruitment of glucose transporters [36]. Intriguingly, this effect does not involve activation of molecules involved in proximal insulin signalling (e.g. PI3K, Akt) and, moreover, no additional enhancement in glucose uptake is observed when muscle cells are simultaneously challenged with the MUFA and a maximally effective concentration of insulin [36]. This latter finding implies that signals initiated by palmitoleate and insulin ultimately converge upon a common end-point that promotes translocation and/or activation of glucose transporters. The current study indicates that, in addition to such effects, incubating myotubes with insulin at a sub-maximal concentration unveils an insulin-sensitising effect of unsaturated fatty acids, such as OA and LOA, which is likely to involve suppression of PP2A action.

Several groups have reported hyperactivation of PP2A in response to sustained oversupply of glucose and SFAs (i.e. glucolipotoxicity), which may be important in the pathogenesis of insulin resistance [22], [37]–[39]. The heightened PP2A activity that is observed under such circumstances may not only involve an increase in PP2Ac expression but also that of the many regulatory PP2A subunits [40], a proposition based on the finding that Ptpa, B55β and B56α are all elevated in muscle and liver of insulin resistant (ZDF) rats [22]. Whilst we cannot discount the possibility that OA and LOA may repress expression of the catalytic and certain regulatory PP2A subunits, our finding that they restrain PA-induced changes in PP2Ac phosphorylation/methylation is likely to be of significance in helping to counter PP2A hyperactivation by SFAs. The differential effect that unsaturated and saturated fatty acids have upon PP2A may be explained, in part, by their contrasting effects on Src, which affects PP2A activity *via* direct phosphorylation of PP2Ac at Tyr^307^ [39], [41]. In line with previous studies [42], we show that PA inhibits Src activation based on a reduction in Src^Y416^ phosphorylation and a concomitant reduction in the downstream phosphorylation of PP2Ac on Tyr^307^ (Fig 6B). As a consequence, PP2Ac would be expected to be more active under these circumstances leading to counter-modulation of signalling molecules activated by insulin. In sharp contrast, as reported in other cell types [43], [44], both OA and LOA induce Scr^Y416^ phosphorylation to a level comparable if not greater than that seen in response to insulin - thus promoting a concomitant increase in PP2Ac tyrosine phosphorylation (Fig 6C, 6D). Unlike PA, the modulation of Src by OA and LOA in our model would suppress PP2A action leading, as we show, to greater and more sustained activation of some of its targets (i.e. Akt and ERK1/2) by insulin. The molecular basis for this differential fatty acid effect on Src is currently unclear but may depend upon their ability to activate membrane bound fatty acid receptors (e.g. GPR40 and GPR120) that couple to signalling molecules influencing the activation status of Src. Activation of such receptors has been implicated in OA-induced activation of ERK1/2 and AP-1 DNA binding activity through a mechanism involving Src in MCF-7 breast cancer cells [43]. Whether such a mechanism explains activation of Src by OA and LOA in skeletal muscle cells and the rather gradual tyrosine phosphorylation and demethylation of PP2Ac is currently unknown, although, we do have evidence for GPR40 expression in L6 myotubes (data not shown). Defining whether GPR40 is a component of the mechanism by which OA and LOA enhance skeletal muscle insulin-sensitivity represents an important investigative goal of future work.

Our results indicate that both OA and LOA promote an increase in proximal insulin signalling and can attenuate the effect that PA has upon Src and PP2Ac phosphorylation. However, it is apparent, from comparing the net changes, that the ability with which both OA and LOA enhance insulin-dependent Akt phosphorylation is somewhat compromised when cells are also incubated with PA (Fig 6A). We recently demonstrated that PA uptake into myotubes is not affected by co-provision of MUFAs and that under these circumstances there is no reduction in PA-derived ceramide synthesis [18]. Consequently, sustained ceramide generation will activate atypical PKCs that physically associate with and negatively regulate Akt activation [7], [8]. However, despite the inability to repress ceramide synthesis from the SFA it is apparent that MUFA and PUFA provision mitigate some of the deleterious effects that PA has upon insulin-action *via*, for example, its ability to activate PP2A.

In summary, the enhanced insulin sensitivity associated with OA and LOA provision is likely to reflect the ability of these unsaturated fatty acids to modulate methylation and phosphorylation of PP2Ac in a manner that represses phosphatase action. Our observations support the view that diets enriched with OA and/or LOA may confer important insulin-sensitising and metabolic benefits. Indeed, recent work assessing dietary fatty acid composition and insulin sensitivity in young adults found that replacing dietary PA with OA benefits clinically relevant measures of metabolic wellbeing, including insulin sensitivity [45]. Although the molecular events underpinning this finding remained unclear, the authors noted that, unlike individuals on an OA-enriched diet, those consuming a PA-enriched diet had more of their fatty acid routed towards acylcarnitine and ceramide production consistent with the idea that PA promotes greater metabolic dysfunction, whereas OA would be not be disruptive in this regard.
